# Biomimetic cell-adhesive ligand-functionalized peptide composite hydrogels maintain stemness of human amniotic mesenchymal stem cells

**DOI:** 10.1093/rb/rbaa057

**Published:** 2021-03-12

**Authors:** Ling Zhang, Na Xiong, Yanfei Liu, Lili Gan

**Affiliations:** Key Laboratory of Cell Engineering of Guizhou Province, Affiliated Hospital of Zunyi Medical University, Zunyi, Guizhou Province, China

**Keywords:** peptide hydrogel, cell-adhesive ligand, human amniotic mesenchymal stem cells, stemness maintenance, osteogenesis differentiation, integrin

## Abstract

*In vivo*, stem cells reside in a three-dimensional (3D) extracellular microenvironment in which complicated biophysical and biochemical factors regulate their behaviors. Biomimicking of the stem cell−matrix interactions is an ideal approach for controlling the stem cell fate. This study investigates the effects of the incorporation of cell-adhesive ligands in 3D self-assembling peptide hydrogels to modulate stem cell survival, proliferation, maintenance of stemness, and osteogenic differentiation. The results show that the composite hydrogels were non-cytotoxic and effective for maintaining human amniotic mesenchymal stem cell (hAMSC) survival, proliferation and phenotypic characterization. The expression levels of pluripotent markers were also upregulated in the composite hydrogels. Under inductive media conditions, mineral deposition and mRNA expression levels of osteogenic genes of hAMSCs were enhanced. The increasing expression of integrin α- and β-subunits for hAMSCs indicates that the ligand−integrin interactions may modulate the cell fate for hAMSCs in composite hydrogels.

## Introduction

MSCs (Mesenchymal stem cells) are mesenchymal-derived pluripotent cells originated from various types of tissues and have been proved an alternative resource for regenerative medicine applications [1, 2]. MSCs can remodel and repair aged, diseased and damaged tissues through direct differentiation [3], paracrine actions [4] and mitochondrial transfer [5]. However, there are still many technical hurdles that need to be overcome as research progresses in this rapidly growing field. For example, culturing or passaging on traditional tissue culture plastic may constrain the expansion potential and differentiation capability of MSCs with increased passage numbers [6]. Furthermore, for many therapeutic applications, steps on guiding the differentiation of the stem cells toward a specific lineage is still a challenge. 

Novel culture systems based on nanofibrous hydrogel may provide a promising opportunity to these challenges [7]. *In vivo*, stem cells reside within a dynamic extracellular microenvironment. The behaviors of stem cells, such as survive, proliferation, differentiation and stemness maintenance are modulated by the complicated microenvironment factors [8]. Stem cells adhere to their surrounding extracellular matrix (ECM) through cell-surface receptors, which can diffuse along the cell membrane and bind to the ligands in ECM via a specific amino acid sequence [9, 10]. These cell-adhesive ligands can be appended to the C-termini of self-assembling peptide sequences directly through solid-phase synthesis and presented as flexible pendent units upon assembly. Such modifications may improve the cellular compatibility, direct desired host cell responses and even endow it with particular functionality [11, 12]. Recent advances suggested that strategies to represent the cell-adhesive ligands in the hydrogels can maintain stemness [13, 14] and guide stem cell differentiation [14−16]. The design and study of such new systems may also be helpful for better understanding of how specific ECM properties contribute to controlling stem cell fate.

**Figure 1.**
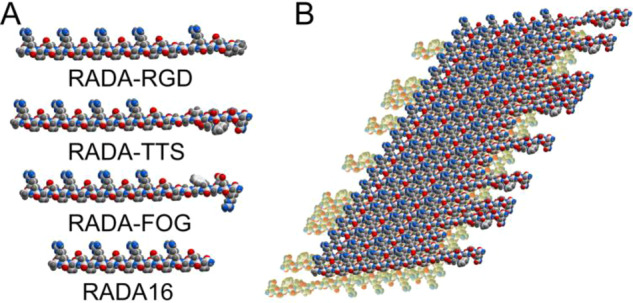
(**A**) Molecular models of RADA-RGD, RADA-TTS, RADA-FOG and RADA16. Atoms are colored by atom type (C, white; N blue; O, red; H gray). (**B**) Schematic model of a self-assembling nanofiber with ligand TTSWSQ (1:1). the designer peptides folded into amphiphilic β-sheets which stacked into a bilayer structure with a hydrophobic core. The incorporating sequences were extending out from the nanofibers.

For this study, cell-adhesive ligands RGDSP, TTSWSQ and GFOGER derived from fibronectin, angiogenesis inducer CCN1 and type I collagen, respectively [17], were chosen and covalently connected to the C-termini of RADA16-I (Ac-RADARADARADARADA-COHN_2_) (Fig. 1A). RADA16-I is able to self-assembly into high aspect ratio nanofibers and spontaneously forms transparent scaffold [18]. Thanks to its biocompatibility, RADA16-I hydrogel can be utilized for regenerative medicine, such as hemostat solutions, tissue recovery, *in vivo* protein/drug delivery platform and 3D cell culture scaffold [19]. One or two glycine residues were employed as spacer between the cell-adhesive ligand and the assembling portion. The incorporating ligands were extending out from the bilayer nanofibers (Fig. 1B). Herein, the influence of various cell-adhesive ligand-bearing self-assembling peptide hydrogels was studied for their effects on human amniotic mesenchymal stem cells (hAMSCs) in terms of the survival, proliferation, maintenance of stemness, as well as osteogenesis differentiation effects. The results show that all of these composite hydrogels were suitable for 3D culture of hAMSCs *in vitro* as a biomimetic scaffold. Importantly, the incorporated ligands involved in cell signaling initiated by cell−matrix interaction, indeed, improved the surface properties of scaffold and influenced the hAMSC behaviors in distinct ways.

## Materials and methods

### Materials

Peptide RADA-RGD, RADA-TTS and RADA-FOG (purity >95%) were commercially synthesized by SciLight Biotechnology LLC. These peptides were blocked with acetyl and amide groups at the N- and C-terminal ends, respectively. High performance liquid chromatography and matrix-assisted laser desorption ionization time-of-flight mass spectrometry analyses of these peptides are shown in Supplementary Figs S2−S4. Peptide RADA16 (purity > 95%) was purchased from Beaver Biosciences, Inc. In brief, the peptide powders were dissolved in deionized water at 1% wt/vol, followed by sonication with an ultrasonic cleaner for 20 min. All the stock solutions were stored at 4°C. The amino sequences of peptide RADA-RGD, RADA-TTS, and RADA-FOG are shown in Fig. S1.

### Transmission electron microscopy

For TEM experiments, the peptides were diluted in deionized water to 0.05% wt/vol using 50 mM PBS (pH 7.2) 1 day before imaging. Then 5 µl of peptide was pipetted on the top of a formvar film-coated grid for negative staining with 1% phosphotungstic acid solution for 1 min. After washing and drying in air, the transmission electron microscope (H-7650, Hitachi) was used to capture the images.

### Circular dichroism

CD (Circular dichroism) spectra of peptide samples diluted in deionized water or 50 mM PBS (pH 7.2) to 0.01% wt/vol were collected on a Chirascan Plus spectrophotometer (Applied photophysics) at 25°C with a bandwidth of 1 nm. The CD spectra of the peptides were analyzed by the SELCON3 program from the CDPro package (Colorado State University), using the SDP48 reference proteins set.

### Fluorescence spectroscopy

The measurements of Thioflavin T (ThT) (Fluorochem) fluorescence were performed using a Fluoromax-4 spectrophotometer (Horiba Scientific) with excitation of 450 nm. Right before test, the peptide solutions were diluted in 50 mM PBS (pH 7.2) to 1.65 mM and incubated at room temperature overnight and then mixed with ThT solutions. The final concentration of ThT was 5 µM.

### Rheology measurement

Time sweep rheology experiments of 1% wt/vol of hydrogel or composite hydrogel were determined using a DHR-2 rheometer (TA Instruments). A cone and plate geometry system (cone diameter 25 mm, angle 1°, truncation gap 51 µM) was used. In brief, 150 µl of mixture which contained the same volume of peptide solution and PBS buffer (pH 7.2) was immediately loaded on the center of the plate. To analyze the elastic characteristic, the mixture was treated with a strain and frequency of 0.5% and 1 Hz for 15 min.

### hAMSCs isolation and characterization

Human placentas were obtained after uncomplicated Caesarean delivery form term pregnancies. Written informed consents were obtained from participants who tested negative for HIV-I and hepatitis virus B and C. The protocol used in the research was assessed and approved by the Medical ethics committee of Zunyi Medical University (ZMUER2018-1-154).

The fresh amniotic membrane was washed in sterile D-Hank’s buffer (containing 1% Penicillin−Streptomycin) until it was totally cleared. Then the membrane was cut into pieces and incubated twice with 0.05% trypsin/0.02% EDTA solution [20]. After gentle agitation at 37°C for 40 min, the remaining tissue was filtered and washed. Then a solution of 0.75 mg/ml of collagenase II (Sigma-Aldrich) and 0.075 mg/ml of DNase I (BioBasic) in DMEM was added. The mixture was gentle agitated at 175 rpm for 1 h to isolate hAMSCs. The isolated cells were cultured with low glucose DMEM (Gibco-BRL) containing 10% FBS (Ausbian), 1% glutaMAX (Gibco-BRL) and 10 ng/100 ml of human FGF basic (R&D Systems) in an incubator at 5% CO_2_. hAMSCs with passage number <5 were used in this study.

To characterize the hAMSCs, the cells suspension was incubated with a cocktail of antibodies containing of CD90-FITC, CD105-PerCP-Cy5.5, CD73-APC, and PE-conjugated anti-CD44, CD45, CD34, CD11b, CD19 and HLA-DR antibodies (BD Biosciences) or the isotype controls. The specific surface antigen phenotypes were examined by a FACSCalibur flow cytometry (BD Biosciences). At last, the expression of vimentin (Sigma-Aldrich) was detected by immunohistochemistry.

### Cell culture in composite hydrogels

The RADA-RGD/TTS/FOG solutions (1% wt/vol) were mixed at a ratio 7:3 with RADA16 solution (1% wt/vol), respectively, and sonicated for 20 min to break the molecular interaction. All the mixed peptide solutions were made at least 2 days prior to the cell encapsulation process. The hAMSCs were harvested, washed and resuspended in 10% sterile sucrose at a density of 2 × 10^6^ cells/ml. Then the equivalent volume of the mixed peptide solution was added to the cell suspension to trigger hydrogel formation. After quickly mixing, the freshly made hydrogel was pipetted into each cell of the multi-well plate before culture medium was added. Depending on the experiments, cells were cultured for 3 − 21 days. The medium was changed regularly. For mineralization experiment and gene expression analysis, the growth medium was replaced with human umbilical cord MSC osteogenic differentiation medium (Cyagen Biosciences, 2 mmol/l β-glycerol-phosphate, 50 µmol/l ascorbic acid, 0.1 µmol/l dexamethasone). The differentiation medium was changed every 3 − 4 days.

### Cell viability and proliferation in composite hydrogel

Fifty microliters of the cell−hydrogel mixture were used per well of a 96-well micro-plate. After 3 days of culture, hAMSCs were labeled using LIVE/DEAD Viability/Cytotoxicity Kit (Molecular Probe) and EdU Labeling/Detection Kit (Ribobio), respectively. In general, the stock solutions of calcein AM and EthD-1 were diluted in PBS according to the manufacturer’s protocol to obtain the working solution. The hydrogels were washed and 100 µl of PBS and 100 µl of the working solution were added sequently. The cells were stained at 37°C for 45 min. After that, the staining solutions were discarded and rinsed gently to remove the free dye form the hydrogels. The percentages of live/dead cells were calculated. The EdU stock solution was diluted to 20 µM with culture media and the cells encapsulated in various hydrogel were incubated in the EdU labeling medium for 18 h at 37°C, followed by fixation with 4% paraformaldehyde for 30 min. The hydrogels were gently washed by PBS for three times and then treated with 100 µl of Apollo reaction cocktail for 30 min at 37°C. After the cells were permeabilized with 0.5% Triton X-100 in PBS, 1× Hoechst 33342 solution was added. The percentage of proliferating cells was calculated.

For CCK-8 assay (Sigma-Aldrich), 50 µl of the cell−hydrogel mixture was used per well of a 96-well plate (*N* = 4). At each interval, the culture medium was changed once followed by addition of CCK-8 agent to the wells at a ratio of 1:10 and incubated for 3.5 h at 37°C. A plate reader (µQuant, BioTek) was employed to measure the absorbance of the 96-well micro-plate at 450 nm. The data were correlated with hydrogel without cells.

### RNA isolation and real-time polymerase chain reaction

At each time point, each cell−hydrogel construct was washed and followed by RNAiso Plus (Takara) extraction (*N* = 3). The total RNA was extracted from the composite hydrogel and 100 ng of total RNA was reverse transcribed using the PrimeScript RT reagent Kit (Takara) into cDNA. RT-PCR was performed with SYBR Premix Ex Taq II (Takara) and the CFX96 Real-Time PCR detection system (Bio-rad). The data were normalized by GAPDH and represented as fold-change over RADA16 group at Day 7. Genes and related specific primers were listed in Supplementary Table S1.

### Alizarin red staining

After 21 days of incubation, the hAMSCs−hydrogel constructs were rinsed twice in PBS and further fixed with 4% paraformaldehyde for 30 min. Then the constructs were stained with 0.35 mg/ml of alizarin red (Sigma-Aldrich) for 20 s and mounted for observation under an inverted optical microscope.

### Quantification of calcium content in the constructs

After 7, 14 or 21 days of incubation, each cell−hydrogel construct was removed from the 24-well plates and washed twice with PBS, incubated with 500 µl lysis buffer (0.25% Triton X-100 in 0.5 M 2-amino-2-methyl-1-propanol). After vortexing, the extracted mixtures were centrifuged and the supernatants were collected for calcium and DNA analysis (*N* = 3). Calcium contents of the encapsulated hMSCs were quantified using a calcium assay kit (Cayman Chemical). The absorbances were measured at 570 nm on a microplate reader. Calcium concentration of each sample was calculated based on the standard curve. DNA contents were also analyzed using a DNA Quantitation Kit (Sigma-Aldrich) and fluorescence intensities of the dye-conjugated DNA solutions were measured using a fluorescence spectrophotometer (Horiba Scientific). All calcium contents were normalized by DNA contents.

### Statistical analysis

Data are expressed as means ± standard deviation (SD). Statistical significance was determined by analysis of variance (one-way ANOVA) with **P < 0.01 or *P < 0.05.

## Results

### Characterization of the functionalized peptides

The secondary structure of the peptide is crucial for the peptides self-assembly. Above a critical concentration, noncovalent interactions, such as hydrophobic and electrostatic forces, π−π hydrogen bonding and van der Waals forces combine to minimize exposure to competitive water molecules and enable hydrogel formation [21, 22]. Thus, the incorporated cell-adhesive sequences that extended from the RADA16 may prevent the self-assembly into nanofibers and hydrogel formation. To address this concern, the characterizations of these functionalized peptides were achieved.

**Figure 2.**
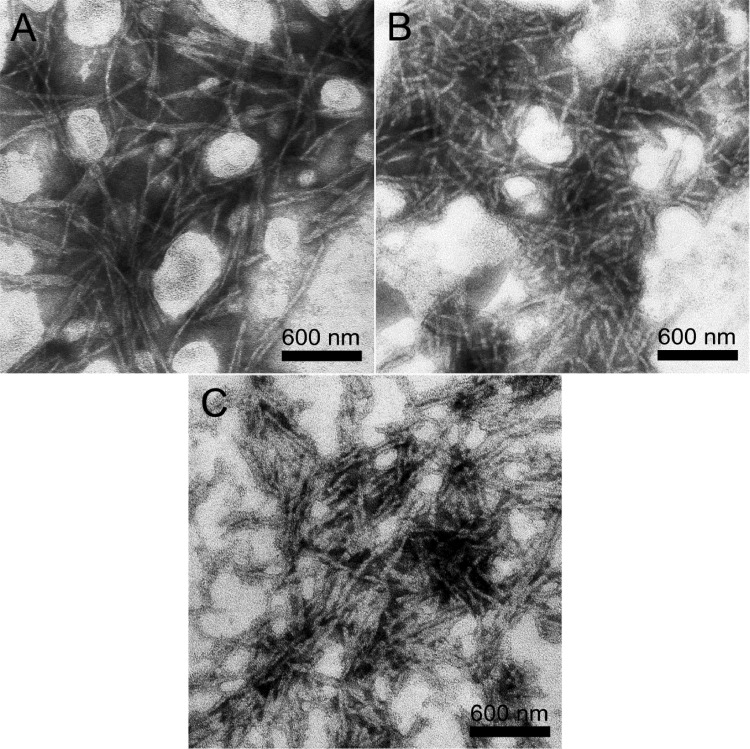
TEM images of the nanostructures formed from (**A**) RADA-RGD, (**B**) RADA-TTS and (**C**) RADA-FOG in phosphate buffer. All of the peptides were diluted with 50 mM PBS (pH 7.2) to 0.05% wt/vol and negatively stained with phosphotungstic acid before observation.

TEM images of the peptide samples in 50 mM phosphate buffer (PBS, pH 7.2) are shown in Fig. 2. All of the functionalized peptides (RADA-RGD, RADA-TTS and RADA-FOG) could self-assemble into well-ordered nanofibers, with widths 20.8 ± 3.5, 24.9 ± 5.6 and 21.5 ± 4.5 nm, respectively, and lengths ranging from hundreds of nanometers to several micrometers. The nanofibers formed by RADA-RGD and RADA-FOG (Fig. 2A and C) were significantly longer than those formed by RADA-TTS (<1 μM, Fig. 2B).

**Figure 3.**
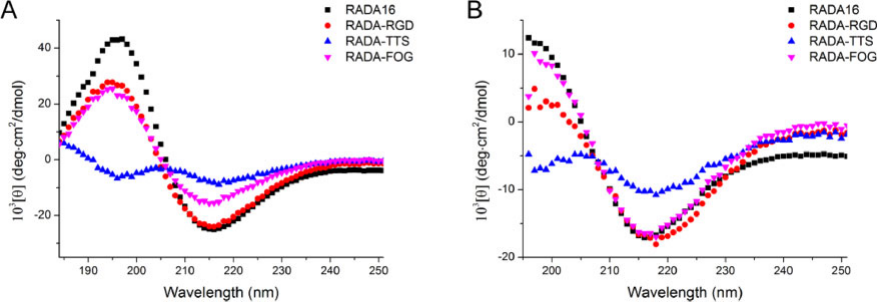
Secondary structures of the peptides RADA-RGD, RADA-TTS, RADA-FOG and RADA16 in (**A**) pure water and (**B**) PBS. The peptides were incubated in pure water or 50 mM PBS (pH 7.2), respectively, to give a final concentration of 0.01% wt/vol. CD spectra were recorded at 25°C.

The CD spectra of 0.01% wt/vol of the functionalized peptides incubated in pure water or PBS (pH 7.2) are shown in Fig. 3. Their CD spectra showed a negative band with a maximum around 217 nm, indicating a high content of β-sheets and a low content of α-helix conformation. The β-sheet contents of RADA-RGD and RADA-FOG were similar to that of RADA16 and the β-sheet content of RADA-TTS was much lower than that of the others. According to the CDpro analysis, the β-sheet contents (Supplementary Table S2; S(r) + S(d)) of RADA-RGD, RADA-TTS, RADA-FOG and RADA16 were 39.4, 34.2, 44.3 and 42.9% in pure water and were 34.8, 30.4, 37.7 and 35.3% in PBS, respectively. Interestingly, the incubation of PBS implies β-structure CD spectra in the 195 nm region. CDpro analysis shows that the β-sheet content of those peptides slightly decreases, while the unordered structures significantly increase (Supplementary Table S2). This change may reflect the structural alteration of the backbone twists of the hydrophobic nanofiber core and the incorporating ligands [23, 24].

**Figure 4.**
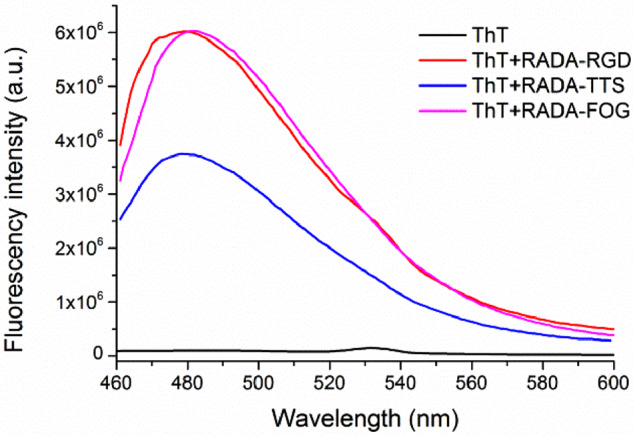
Fluorescence spectra of ThT in PBS with peptide RADA-RGD, RADA-TTS and RADA-FOG. The measurements of ThT fluorescence were recorded at 450 nm excitation. Before measurement, the peptide solutions were diluted with 50 mM PBS (pH 7.2) to 1.65 mM and incubated at room temperature overnight and then mixed with ThT solutions. The final concentration of ThT was 5 µM.

This observation was confirmed by the ThT binding assay that has been used for studying the β-sheet formation of amyloid-like fibrils. ThT did not produce the characteristic fluorescent signal alone; the fluorescence appears with the maximum at 480 nm upon addition of nanofibers, leading to RADA-RGD, RADA-TTS and RADA-FOG (Fig. 4). The dramatic increases in fluorescence emission confirm the β-sheet structure of these peptides [25]. Furthermore, RADA-TTS had a significantly lower fluorescence peak than the others. This observation was consistent with the CD data that RADA-TTS had the lowest β-sheet content (Fig. 3; Supplementary Table S2). Since ThT is reported to bind as a monomer on the cross β-sheet surfaces along the long axis of amyloid-like fibrils [26]. According to the ThT binding model proposed in [27], the lower β-sheet content of the RADA-TTS led to a decrease of the number of ThT binding sites on the nanofibers, and thus a decrease of the fluorescence intensity.

Rheological measurements were performed to test the gel formation abilities of the peptides. In accordance with the above observations, the hydrogel formation process of 1% wt/vol of RADA-RGD and RADA-FOG occurred in a few minutes once they were mixed with an equal volume of PBS (pH 7.2). The storage modulus of the hydrogel increased as the time goes on, whereas the volume of loss modulus did not change with time (Supplementary Fig. S5). The increasing storage modulus reflects the elongation of the nanofibers under self-assembling conditions. In contrast, peptide RADA-TTS behaved like a liquid and failed to form a self-supporting hydrogel.

These data indicate that the functionalized sequences, indeed, influence the self-assembly and hydrogel formation. To increase the mechanical strength of these soft hydrogels, the functionalized peptide solutions (1% wt/vol) were individually mixed at a ratio of 7:3 with RADA16 (1% wt/vol) and sonicated for 20 min. The mixed solutions were held at room temperature for at least 2 days to ensure nanofiber network formation. The RADA16 peptide was integrated into the individual RADA-RGD, RADA-TTS and RADA-FOG nanofibers as presented in the molecular model (Fig. 1B). The composite hydrogels (abbreviated as RGDmix, TTSmix and FOGmix, respectively) were also subjected to rheological analysis. The mechanical strength was greatly increased for all of the peptide mixtures (Supplementary Fig. S6), and each composite peptide self-assembled into a much more structurally stable hydrogel without producing any obvious changes in the physical appearance. The composite hydrogels can be handled and transported more easily and then used for the following purposes.

### Characterization of hAMSCs

**Figure 5.**
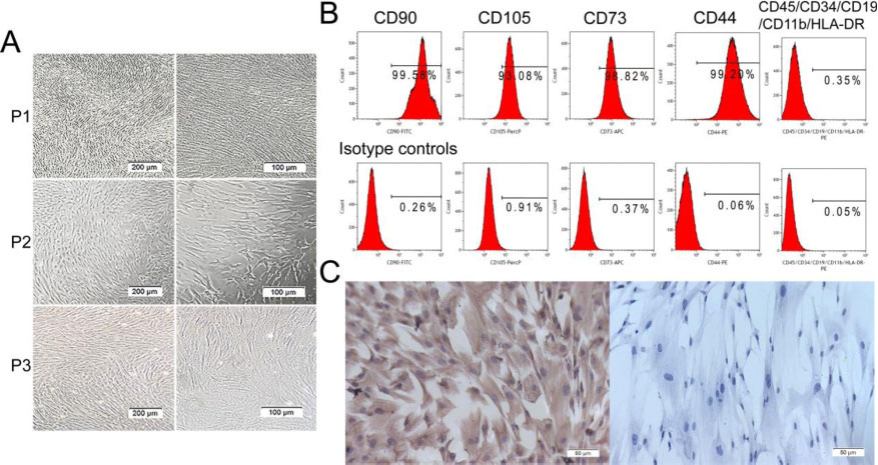
Morphology and characterization of human amniotic mesenchymal stem cells (hAMSCs). (**A**) morphology of passage 1−passage 3 hAMSCs. (**B**) Expression of hAMSCs surface markers. hAMSCs were positive for CD90, CD105, CD73, CD44 and negative for CD45, CD34, CD19, CD11b and HLA-DR. (**C**) Expression of vimentin of hAMSCs by immunohistochemistry.

The hAMSCs were isolated from human amnions by enzymatic digestion. The passage 1 − 3 cells exhibited a typical adherent cell monolayer with a uniformly spindle, fibroblast-like morphology in a swirling arrangement (Fig. 5A). Flow cytometry indicated that hAMSCs highly positive for the mesenchymal stem cell (MSCs) surface markers CD90, CD105, CD73 and CD44, but negative for hematopoietic markers such as CD45, CD34, CD19 and CD11b as well as class II antigen HLA-DR (Fig. 5B). Furthermore, immunohistochemical staining revealed that hAMSCs highly expressed MSC surface marker vimentin (Fig. 5C). These findings agree with the criteria for MSCs identification by The Association of International Cell Therapy [28].

### hAMSCs proliferation in composite hydrogels

A suitable bioactive scaffold should provide an optimal environment for the cell to survive, grow, and achieve the desired cellular functions. Thus, the potential of the composite hydrogels as biomimetic scaffolds was tested. hAMSCs were encapsulated into the composite hydrogels and these cells adopted a spread morphology after hours of culture (Supplementary Fig. S7). After 1 day, the viability of hAMSCs was measured by live/dead staining, in which live cells fluoresce green and dead cells fluoresce red. Supplementary Fig. S8 shows that >96% of the encapsulated hAMSCs were alive (without statistical significance), indicating that the composite hydrogels are non-cytotoxic and effective for maintaining cell survival.

**Figure 6.**
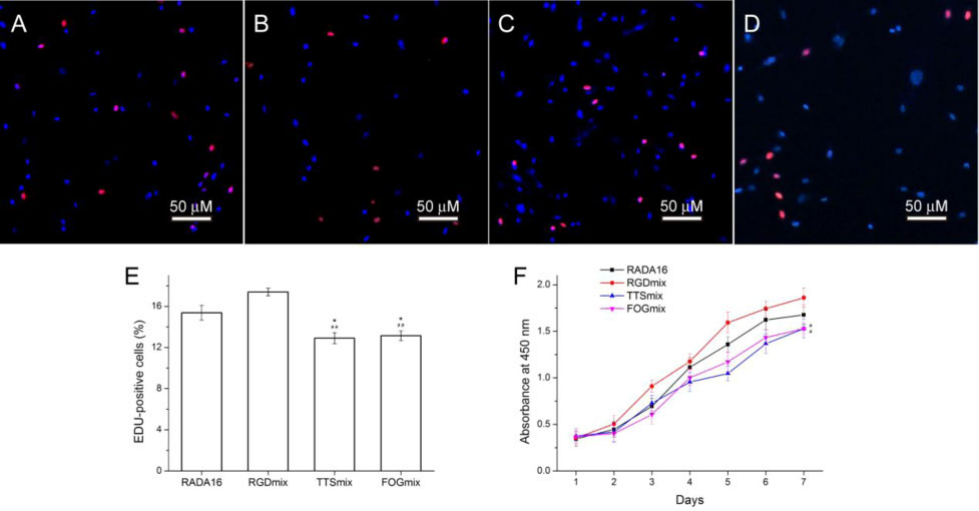
EdU Staining of hAMSCs encapsulated in hydrogels (**A**) RADA16, (**B**) RGDmix, (**C**) TTSmix and (**D**) FOGmix after 3 days of culture (red: EdU; blue: Hoechst 33342 for nuclei staining); and (**E**) percentage of EdU-positive hAMSCs (%). 15.37 ± 0.71, 17.40 ± 0.39, 12.91 ± 0.53 and 13.14 ± 0.46% of the hAMSCs encapsulated in RADA16, RGDmix, TTSmix and FOGmix were positive for EdU, respectively. *P < 0.05 compared with RADA16, ^##^*P* < 0.01 compared with RGDmix. All data shown are means ± SD of three independent experiments. (**F**) Proliferation curves of cultured hAMSCs as determined by the CCK-8 test. RGDmix: RADA-RGD+RADA16, TTSmix: RADA-TTS+RADA16, FOGmix: RADA-FOG+RADA16 (all mixture ratio is 7:3). individual RADA16 hydrogel (1% wt/vol) was used as control.

hAMSC proliferation encapsulated in various composite hydrogels were detected via EdU staining and CCK-8 assays. EdU can readily incorporate into cellular DNA during the S phase of the cell cycle, and it has been successfully employed in the detection of cell proliferation [29]. Figure 6A−E shows that hAMSCs cultured in an RGDmix hydrogel had the highest EdU-positive rate among different composite hydrogel groups, suggesting that the hAMSCs spend less time on adapting to the hydrogel environment. It is not surprisingly that the fibronectin-derived RGDSP sequence stimulates various integrins and cell proliferation [17]. The EdU-positive rate of cell cultured in TTSmix (12.91 ± 0.53%) and FOGmix hydrogel (13.14 ± 0.46%) was lower than that of cells cultured in RADA16 (15.37 ± 0.71%) and RGDmix hydrogel (17.40 ± 0.39%). To further confirm this result, we performed a CCK-8 proliferation assay. Consistent with the EdU staining results, the number of hAMSCs encapsulated in RGDmix hydrogel was significantly higher than the other groups after 7 days of culture. The hAMSCs in TTSmix and FOGmix hydrogel still had a slower proliferation rate than that for RGDmix hydrogel. These results suggested that the proliferation of hAMSCs was varied by the functionalized hydrogels. This may be due to the various ligands that recognize and bind to specific integrins influencing the proliferation of hAMSCs in different ways.

### Stemness of hAMSCs embedded in composite hydrogels

**Figure 7.**
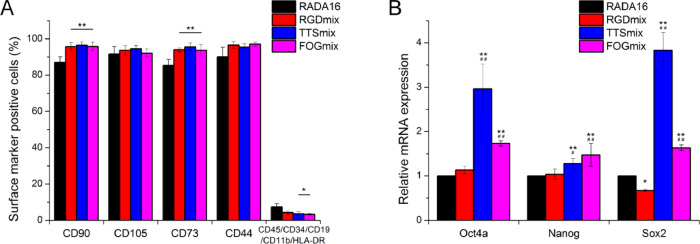
Composite hydrogels allow long-term maintenance of stemness of hAMSCs. (**A**) Expression of hAMSCs surface markers in composite hydrogels. (**B**) mRNA expressions of stemness-associated genes were measured after 3 days of culture in composite hydrogels. The relative expression was analyzed by the 2^-ΔΔCt^ method, with normalization by the Ct of GAPDH and calibrated to RADA16 group. *P < 0.05, **P < 0.01 compared with RADA16. ^##^*P* < 0.01 compared with RGDmix. All data shown are means ± SD of three independent experiments. RGDmix: RADA-RGD+RADA16, TTSmix: RADA-TTS+RADA16, FOGmix: RADA-FOG+RADA16 (all mixture ratio is 7:3).

To maintain stemness, stem cells should constantly express proteins characteristic of their stem cell state. Here, the phenotypic characterizations of hAMSCs embedded in various composite hydrogels were performed after 28 days of culture in noninductive media without passage. Figure 7A and Supplementary Table S3 show that the percentages of CD90 and CD73 positive hAMSCs seeded in RGDmix, TTSmix and FOGmix were significantly higher than that in RADA16, while the percentage of cells in RADA16 hydrogel positive for the hematopoietic markers CD45/CD34/CD19/CD11b/HLA-DR was significantly higher than that in the TTSmix and FOGmix. These results suggested that more hAMSCs seeded in RADA16 hydrogel had lost their stemness features and differentiated into multiple lineages.

Pluripotent markers, such as Oct4a, Nanog and Sox2, have been identified in hAMSCs. They play an important role in self-renewal and differentiation capabilities of hAMSCs [6]. To further investigate the changes in the stem cell characteristics of hAMSCs embedded within the composite hydrogel microenvironment compared with hAMSCs in the RADA16 hydrogel (Fig. 7B), the relative expression levels of stemness-associated genes were analyzed (via mRNA). Real-time PCR analysis showed that the relative mRNA expression of Oct4a, Nanog and Sox2 in hAMSCs seeded in the TTSmix and FOGmix hydrogels were significantly higher than those seeded in the RADA16 hydrogel, and the RGDmix hydrogel failed to promote the expression of the pluripotency-associated genes compared with the RADA16 hydrogel.

### Osteogenesis differentiation of hAMSCs in composite hydrogels

**Figure 8.**
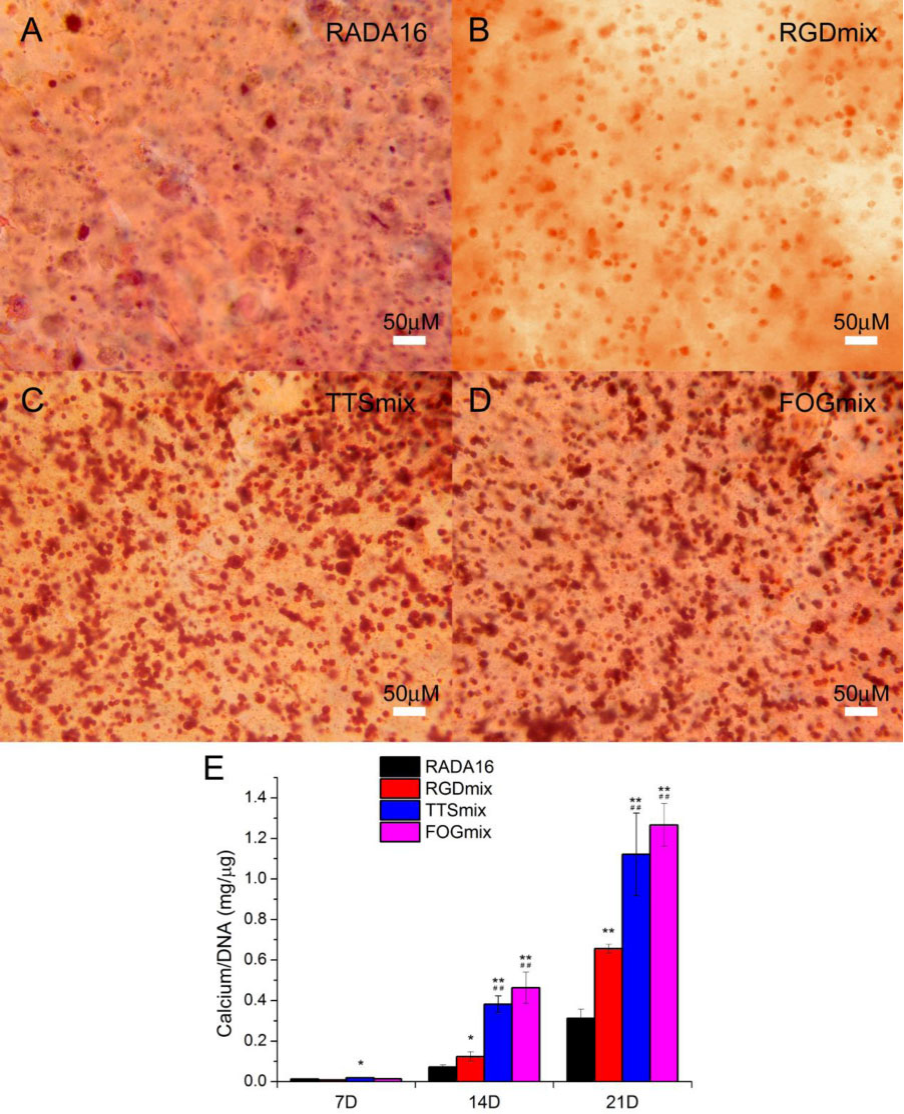
Functionalized self-assembling peptide hydrogels promoted hAMSCs mineralization. (**A**−**D**) Alizarin red staining images of hAMSCs−hydrogel constructs after 3 weeks of culture in osteogenic medium. (**E**) Quantification of calcium deposits within various constructs. All calcium content measurements were normalized by DNA content within each construct. *P < 0.05, **P < 0.01 compared with RADA16. ^##^*P* < 0.01 compared with RGDmix. All data shown are means ± SD of three independent experiments. RGDmix: RADA-RGD+RADA16, TTSmix: RADA-TTS+RADA16, FOGmix: RADA-FOG+RADA16 (all mixture ratio is 7:3).

To evaluate the effect of functionalized sequences on the osteogenesis differentiation of hAMSCs, the cell−hydrogel constructs were submerged in commercial osteogenesis differentiation medium for up to three weeks. The extent of mineralized ECM formed in the various composite hydrogels was examined by Alizarin Red staining after 3 weeks of incubation in the differentiation medium (Fig. 8A−D). TTSmix and FOGmix hydrogels showed intense Alizarin red staining compared with RADA16 and RGDmix hydrogels, indicating that more calcium was deposited in the former. The calcium content was significantly increased in RGDmix hydrogels (*P* < 0.05) and TTSmix and FOGmix hydrogels (*P* < 0.01) compared with RADA16 after 14 days (Fig. 8E). TTSmix and FOGmix hydrogels gave the highest values, and thus, led to more calcium deposition. The calcium content in all of each hydrogel continued to increase with time. The capacity to accumulate calcium in the ECM proved that the hAMSCs encapsulated into the hydrogels differentiated into mature osteoblasts that could deposit calcium in the ECM.

**Figure 9.**
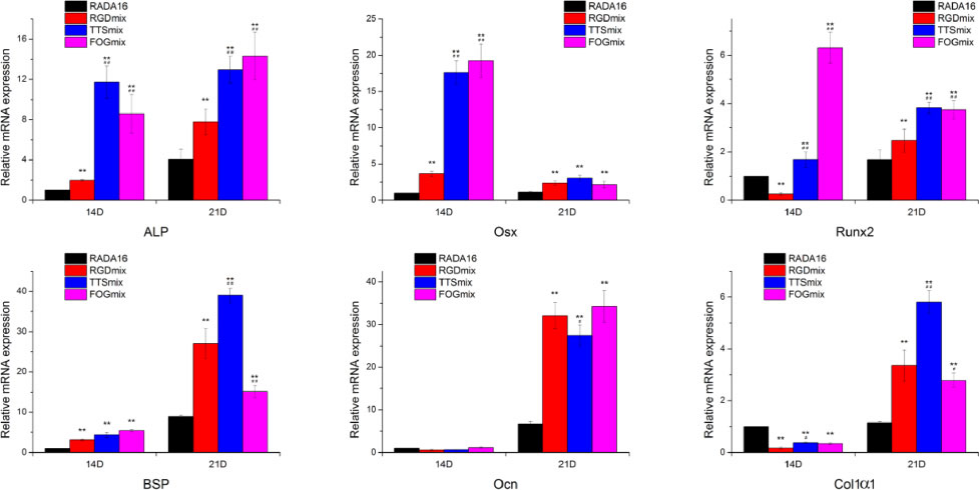
Osteogenic gene expression of hAMSCs cultured in various hydrogels. The relative expression was analyzed by the 2^-ΔΔCt^ method, with normalization by the Ct of GAPDH and calibrated to RADA16 group on Day 7. *P < 0.05, **P < 0.01 compared with RADA16. ^#^*P* < 0.05, ^##^*P* < 0.01 compared with RGDmix. All data shown are means ± SD of three independent experiments. RGDmix: RADA-RGD+RADA16, TTSmix: RADA-TTS+RADA16, FOGmix: RADA-FOG+RADA16 (all mixture ratio is 7:3).

We further estimated the osteogenic gene expression of hAMSCs cultured in various hydrogels on Days 14 and 21 by qRT-PCR (Fig. 9) to determine the extent of the differentiation process. Alkaline phosphatase (ALP), osterix (Osx), type I collagen (Col1α1) and runt-related transcription factor 2 (Runx2) are early-stage markers of osteogenic differentiation of MSC while bone sialoprotein (BSP) and osteocalcin (Ocn) are later ones. Almost all of the osteogenic gene expression data were significantly increased after 2 − 3 weeks incubation in the differentiation medium. The expression levels of Runx2 and ALP were significantly upregulated when hAMSCs were cultured in composite hydrogels compared with the hAMSCs in RADA16 on days 14 and 21; the expression of Runx2 and ALP could more rapidly increase in TTSmix and FOGmin hydrogels. The maximum Osx expression was observed on day 14 for all of the hAMSCs−hydrogel constructs and was dramatically decreased at day 21. The expression levels of Osx in TTSmix and FOGmin hydrogels were significantly higher than that in the RGDmix hydrogel. After 21 days of incubation in osteogenesis differentiation medium, the BSP and Col1α1 genes showed maximum expression in all of the hAMSCs−hydrogel constructs, while the expression levels of both genes in the RGDmix hydrogel were significantly lower than that in the TTSmix hydrogel, but higher than that in the FOGmix hydrogel. All of the composite hydrogels could also increase the BSP gene expression on day 14, but a reversal was seen for the Col1α1 gene. The expression levels of Ocn in the functionalized peptide hydrogels were higher than those in the RADA16 group at day 21, and there was no significant difference between groups at day 14. These results indicate that the incorporation of cell-adhesive ligands upregulated the mineralization process to varying extents.

## Discussion

MSCs can be derived from multiple tissue sources including bone marrow, adipose tissue, peripheral blood [1, 2]. Among them, hAMSCs which isolated from human amnions have the potential to be used in many areas of regenerative medicine because of their high plasticity, viability, ultralow immunogenicity and tumorigenicity [30]. Moreover, hAMSCs can be acquired non-invasively and do not involve medical ethical or legal problems, because amniotic membrane is a waste product of perinatal tissue sources, which is routinely discarded after delivery [20, 31]. Therefore, hAMSCs have been successively serviced as a viable resource to treat diseases, such as neurological disorders [32], liver [33] and lung injury [34], chronic wound [35], and acute inflammatory diseases [36], and exhibit great potential in bone and osteochondral regeneration [31, 37].

MSCs isolation and expansion can be efficiently performed on traditional petri dishes, but the *in vitro* 2D culture will alter their behaviors and result in constant senescence [38], a rapid loss of MSC phenotype and stemness, and decreased differentiation capacity [6, 39], Thus, only hAMSCs at low passage numbers (less than 5) were used for *in vitro* or *in vivo* experiments. Both regenerative medicine approaches to construct functional tissue replacements form stem cell and clinical transplantation require a large number of functional cells [40]. Overcoming this limitation relies on constructing well-defined cell culture platforms for stem cell expansion and directed differentiation.

Key elements, such as ECM composition, architecture, cell−ECM interactions, cell–cell interactions and mechanical signals, contribute to the regulation of the stem cell by niche [41]. Thus, closely mimicking the stem cell niche may provide a plausible approach to enhancing the efficiency expansion and directed differentiation of stem cells [40]. Engineered hydrogel scaffold properties, including stiffness, composition, microstructure, degradation rate, and cell-scaffold adhesion, mediated by integrins could maintain stemness [13, 40, 42]. Thus, the long-term maintenance of the MSC phenotype and upregulated expression of pluripotent markers could be interpreted in at least two ways. First, the functionalized cell-adhesive ligands RGDSP, TTSWSQ and GFOGER that present to the encapsulated hAMSCs at high density bind to distinct integrin receptors and can elaborate the stem cell−matrix interactions by activating various signaling pathways [43]. Mimicking the native tissue microenvironment with high density of cell-adhesive ligands can contribute to the promotion of stem cell viability and proliferation. This, in turn, optimizes the ligation of integrins α5β1, αvβ5, α6β1 and α9β1 through a combination of multiple adhesion peptides. The functionalized poly(ethylene glycol) (PEG) hydrogel could strongly upregulate the mRNA expression of stemness-related genes, including Oct4a, Nanog, and Sox2 [13, 42]. Meanwhile, an immobilized laminin-derived pentapeptide, YIGSR, can promote adhesion and survival of the MSCs, as well as the expression of Oct4a, Nanog and Sox2 in these cells [44]. Second, an increase in the pluripotent marker expression may also be interpreted by the hydrogel stiffness. Cells mechanically adhere to the ECM through integrins via specific adhesive ligands presented by ECM proteins. At focal contacts, integrins mediate the physical linkage between the ECM and the intracellular actin fibers of a cell. They activate various signaling pathways that ultimately result in changes to gene expression [45]. By mimicking the ECM mechanical stiffness, nanofibrous biomaterials provide stem cells with 3 D environment to maintain the stem cell stemness. For instance, studies have shown that embryonic stem cell cultured on soft polyacrylamide gels (∼600 Pa) have increased self-renewal [46, 47]. Soft dextran-based hydrogels (∼250 Pa) strengthen the expression of Oct4a, Nanog and Sox2 in adipose-derived stem cells [48]. In our work, all of the composite hydrogels are quite compliant (*G*′<650 Pa; Supplementary Fig. S6A) compared with most of the gels formed by natural or synthetic materials. The most compliant composite hydrogel (TTSmix) exhibited a significantly enhanced stemness biomarkers expression, while these biomarkers were unchanged for the stiffest hydrogel (RGDmix). Both the cell-adhesive ligands and the hydrogel compliant stiffness may have a synergistic effect on the enhanced stemness and biomarker expression.

Differentiated cells rather than stem cells are required for many cell-based regenerative medicine applications. Designing an engineered culture platform to guide the differentiation of stem cells to a desired linkage may facilitate the translation of MSCs from laboratory to clinic. Many of the same niche factors that promote stemness may also enhance stem cell differentiation capabilities [49, 50]. Thus, we evaluated the osteogenesis differentiation capability of hAMSCs under differentiation medium. As expected, the hAMSCs that exhibited higher stemness characteristics were induced more easily to differentiate into an osteogenic lineage. The results suggest that the increasing osteogenesis differentiation may be attributed to the enhanced expression of pluripotent factors because they exert a great effect on both renewal and differentiation capabilities. Meanwhile, the identity of cell-adhesive ligands could also regulate the stem cell differentiation. For instance, both the laminin mimetic IKVAV (when presented in combined with RGD sequences) [43] and collagen-derived ligands DGEA [15] and GFOGER [51, 52] could promote the MSCs osteogenic differentiation. The incorporation of RGD-containing peptides will enhance osteogenesis and matrix mineralization of BMSCs cultured in the hydrogels [53–55].

**Figure 10.**
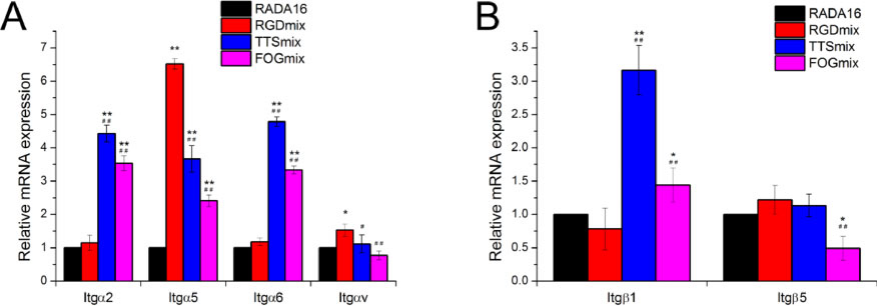
Composite hydrogels promoted mRNA expression of integrin subunits in hAMSCs. The mRNA expression of (**A**) integrin α subunits and (**B**) integrin β subunits were measured. The relative expression was analyzed by the 2^-ΔΔCt^ method, with normalization by the Ct of GAPDH and calibrated to RADA16 group. *P < 0.05, **P < 0.01 compared with RADA16. ^##^*P* < 0.01 compared with RGDmix. All data shown are means ± SD of three independent experiments. RGDmix: RADA-RGD+RADA16, TTSmix: RADA-TTS+RADA16, FOGmix: RADA-FOG+RADA16 (all mixture ratio is 7:3).

To further estimate whether the adhesive ligands influence the maintenance of stemness and enhancing differentiation capabilities, we next measured the mRNA expression of integrin α2, α5, α6, αv, β1 and β5 subunits (Fig. 10). Increased expression was seen for α2 and αv for RGDmix and for α2, α5, α6 and β1 for TTSmix and FOGmix, respectively. The cell−ECM interaction is important for the modulation of stem cell fate and is mediated by the binding between the integrins and adhesive ligands [8, 43]. Each cell-adhesive ligand can be recognized by various integrins. Specifically, the RGDSP sequence can be recognized by multiple integrins, including integrin α5β1 and αvβ5 [42]. The sequences TTSWSQ and GFOGER bind specifically to integrin α6β1 and α2β1, respectively [42, 52, 56]. The binding between these ligands and these counter integrin receptors elicits signals that are transmitted into the cell, which induces a vast number of structural and signaling changes within the cell and ultimately results in the integrin-dependent regulation of cell behaviors, e.g. survival, adhesion, proliferation, migration, stemness and differentiation [8, 45]. More detailed studies of these possible pathways should be conducted to have a better understanding of the interactions between stem cells and the mimicking environments. In addition, physical cues, such as mechanical simulation, nanofiber alignment and peptide motifs, lead to changes in integrin expression. Upregulation of various integrin α- and β-subunits have also been observed for embryonic stem cells on poly(l-lactic acid) scaffolds [57]. Encapsulation in RADA16 hydrogels could promote the expression of integrin α2, α4, α5 and αv, as well as β1 subunits, for both MSCs and hematopoietic stem cells by mimicking key features of marrow physiology [58]. The RGD peptide bearing a PEG hydrogel was found to upregulate the survival and αvβ3 and α4 production of hMSCs [59]. The mechanical simulation and aligned features of nanofibers composed of poly(lactide-*co*-glycolide) may also contribute to an enhanced expression of α2, α5 and β1 subunits [60]. The α5 integrin complexes with a β1 subunit in human MSCs is upregulated by binding to the poly(amidoamine) functionalized surfaces [61]. The elevated expression of these integrin subunits is likely due to the natural response for cell seeding in a 3D biomimicking environment because more integrin subunits are required to ensure the development of mature focal contact assembly and signal transduction from outside to inside.

## Conclusion

Overall, hydrogels incorporated with three individual cell-adhesive ligands could preserve the hAMSCs phenotype, promote the expression of pluripotent markers, and enhance the osteogenesis differentiation and matrix mineralization under induction conditions to different extent. The varying upregulation of integrin α and β subunits suggests that distinct integrin mediated pathways may be involved in the fate regulation of hAMSCs. Mechanical simulation may also play a synergetic effect with the cell-adhesive ligands on the modulation of stem cell fate. Our findings provide clues for designing new cell-adhesive ligands bearing materials that regulate stem cell fate. These results will guide further design of multifunctional hydrogel materials for biomedical applications.

## Supplementary Material

rbaa057_Supplementary_Data

## References

[1] Brown C , McKee C, Bakshi S et al Mesenchymal stem cells: cell therapy and regeneration potential. J Tissue Eng Regen Med 2019;13:1738–55. 31216380 10.1002/term.2914

[2] Tsuno H , Yoshida T, Nogami M et al Application of human amniotic mesenchymal cells as an allogeneic transplantation cell source in bone regenerative therapy. Mater Sci Eng C 2012;32:2452–8.

[3] Cohen DE , Melton D. Turning straw into gold: directing cell fate for regenerative medicine. Nat Rev Genet 2011;12:243–52. 21386864 10.1038/nrg2938

[4] Liang X , Ding Y, Zhang Y et al Paracrine mechanisms of mesenchymal stem cell-based therapy: current status and perspectives. Cell Transplant 2014;23:1045–59. 23676629 10.3727/096368913X667709

[5] Paliwal S , Chaudhuri R, Agrawal A et al Regenerative abilities of mesenchymal stem cells through mitochondrial transfer. J Biomed Sci 2018;25:31. 29602309 10.1186/s12929-018-0429-1 PMC5877369

[6] Ghamari S-H , Abbasi-Kangevari M, Tayebi T et al The bottlenecks in translating placenta-derived amniotic epithelial and mesenchymal stromal cells into the clinic: current discrepancies in marker reports. Front Bioeng Biotechnol 2020;8:180. 32232037 10.3389/fbioe.2020.00180 PMC7083014

[7] Chan KH , Lee WH, Ni M et al C-terminal residue of ultrashort peptides impacts on molecular self-assembly, hydrogelation, and interaction with small-molecule drugs. Sci Rep 2018;8:17127. 30459362 10.1038/s41598-018-35431-2 PMC6244206

[8] Nicolas J , Magli S, Rabbachin L et al 3D extracellular matrix mimics: fundamental concepts and role of materials chemistry to influence stem cell Fate. Biomacromolecules 2020;21:1968–94. 32227919 10.1021/acs.biomac.0c00045

[9] Discher DE , Mooney DJ, Zandstra PW. Growth factors, matrices, and forces combine and control stem cells. Science 2009;324:1673–7. 19556500 10.1126/science.1171643 PMC2847855

[10] Guilak F , Cohen DM, Estes BT et al Control of stem cell fate by physical interactions with the extracellular matrix. Cell Stem Cell 2009;5:17–26. 19570510 10.1016/j.stem.2009.06.016 PMC2768283

[11] Liu Y , Yang Y, Wang C et al Stimuli-responsive self-assembling peptide made from antibacterial peptide. Nanoscale 2013;5:6413–21. 23739953 10.1039/c3nr00225j

[12] Wei W , Liu Y, Xiong N et al A peptide-based method for the fabrication of 1D rail-like nanoparticle chains and 2D nanoparticle membranes: higher-order self-assembly. ChemPlusChem 2019;84:374–81. 31939204 10.1002/cplu.201900040

[13] Lee ST , Yun JI, Vlies AJVD et al Long-term maintenance of mouse embryonic stem cell pluripotency by manipulating integrin signaling within 3D scaffolds without active Stat3. Biomaterials 2012;33:8934–42. 22998814 10.1016/j.biomaterials.2012.08.062

[14] Hilderbrand AM , Ovadia EM, Rehmann MS et al Biomaterials for 4D stem cell culture. Curr Opin Solid State Mater Sci 2016;20:212–24. 28717344 10.1016/j.cossms.2016.03.002 PMC5510611

[15] Mehta M , Madl CM, Lee S et al The collagen I mimetic peptide DGEA enhances an osteogenic phenotype in mesenchymal stem cells when presented from cell-encapsulating hydrogels. J Biomed Mater Res 2015;103:3516–25. 10.1002/jbm.a.35497 PMC4589437 25953514

[16] Tong X , Yang F. Sliding hydrogels with mobile molecular ligands and crosslinks as 3D stem cell Niche. Adv Mater 2016;28:7257–63. 27305637 10.1002/adma.201601484 PMC5127628

[17] Barker TH. The role of ECM proteins and protein fragments in guiding cell behavior in regenerative medicine. Biomaterials 2011;32:4211–4. 21515169 10.1016/j.biomaterials.2011.02.027

[18] Wang X , Wang J, Guo L et al Self-assembling peptide hydrogel scaffolds support stem cell-based hair follicle regeneration. Nanomed Nanotechnol Biol Med 2016;12:2115–25. 10.1016/j.nano.2016.05.021 27288668

[19] Zhang S , Gelain F, Zhao X. Designer self-assembling peptide nanofiber scaffolds for 3D tissue cell cultures. Semin Cancer Biol 2005;15:413–20. 16061392 10.1016/j.semcancer.2005.05.007

[20] Liu R , Zhang X, Fan Z et al Human amniotic mesenchymal stem cells improve the follicular microenvironment to recover ovarian function in premature ovarian failure mice. Stem Cell Res Ther 2019;10:299. 31578152 10.1186/s13287-019-1315-9 PMC6775662

[21] Liu Y , Zhang L, Wei W. Effect of noncovalent interaction on the self-assembly of a designed peptide and its potential use as a carrier for controlled bFGF release. Int J Nanomed 2017;17:659–70. 10.2147/IJN.S124523 PMC5261598 28176898

[22] Mart RJ , Osborne RD, Stevens MM et al Peptide-based stimuli-responsive biomaterials. Soft Matter 2006;2:822–35. 32680274 10.1039/b607706d

[23] Zhang S , Rich A. Direct conversion of an oligopeptide from a β-sheet to an α-helix: a model for amyloid formation. Proc Natl Acad Sci USA 1997;94:23–38. 8990154 10.1073/pnas.94.1.23 PMC34557

[24] Mishra A , Chan KH, Reithofer MR et al Influence of metal salts on the hydrogelation properties of ultrashort aliphatic peptides. RSC Adv 2013;3:9985–93.

[25] Bagrov D , Gazizova Y, Podgorsky V et al Morphology and aggregation of RADA-16-I peptide studied by AFM, NMR and molecular dynamics simulations. Biopolymers 2016;106:72–81. 26501800 10.1002/bip.22755

[26] Sidhu A , Vaneyck J, Blum C et al Polymorph-specific distribution of binding sites determines thioflavin-T fluorescence intensity in α-synuclein fibrils. Amyloid 2018;25:189–96. 30486688 10.1080/13506129.2018.1517736

[27] Biancalana M , Koide S. Molecular mechanism of thioflavin-T binding to amyloid fibrils. Biochim Biophys Acta 2010;1804:1405–12. 20399286 10.1016/j.bbapap.2010.04.001 PMC2880406

[28] Dominici M , Blanc KL, Mueller I et al Minimal criteria for defining multipotent mesenchymal stromal cells. The International Society for Cellular Therapy position statement. Cytotherapy 2006;8:315–7. 16923606 10.1080/14653240600855905

[29] Pereira PD , Serra-Caetano A, Cabrita M et al Quantification of cell cycle kinetics by EdU (5-ethynyl-2'-deoxyuridine)-coupled-fluorescence-intensity analysis. Oncotarget 2017;8:40514–32. 28465489 10.18632/oncotarget.17121 PMC5522303

[30] Syva SH , Ampon K, Lasimbang H et al Microenvironmental factors involved in human amnion mesenchymal stem cells fate decisions. J Tissue Eng Regen Med 2017;11:311–20. 26073746 10.1002/term.2043

[31] You Q , Liu Z, Zhang J et al Human amniotic mesenchymal stem cell sheets encapsulating cartilage particles facilitate repair of rabbit osteochondral defects. Am J Sports Med 2020;48:599–611. 31940211 10.1177/0363546519897912

[32] Yu SJ , Soncini M, Kaneko Y et al Amnion: a potent graft source for cell therapy in stroke. Cell Transplant 2009;18:111–8. 19499700 10.3727/096368909788341243

[33] Manuelpillai U , Tchongue J, Lourensz D et al Transplantation of human amnion epithelial cells reduces hepatic fibrosis in immunocompetent CCl_4_-treated mice. Cell Transplant 2010;19:1157–68. 20447339 10.3727/096368910X504496

[34] Moodley Y , Ilancheran S, Samuel C et al Human amnion epithelial cell transplantation abrogates lung fibrosis and augments repair. Am J Respir Crit Care Med 2010;182:643–51. 20522792 10.1164/rccm.201001-0014OC

[35] Prakoeswa CRS , Natallya FR, Harnindya D et al The efficacy of topical human amniotic membranemesenchymal stem cell-conditioned medium (hAMMSC-CM) and a mixture of topical hAMMSC-CM + vitamin C and hAMMSC-CM + vitamin E on chronic plantar ulcers in leprosy: a randomized control trial. J Dermatolog Treat 2018;29:835–40. 29671368 10.1080/09546634.2018.1467541

[36] Yamahara K , Hamada A, Soma T et al Safety and efficacy of amnion-derived mesenchymal stem cells (AM01) in patients with steroid-refractory acute graft-versus-host disease after allogeneic haematopoietic stem cell transplantation: a study protocol for a phase I/II Japanese trial. BMJ Open 2019;9:e026403. 10.1136/bmjopen-2018-026403 PMC6615811 31289066

[37] Tsuno H , Yoshida T, Nogami M et al Application of human amniotic mesenchymal cells as an allogeneic transplantation cell source in bone regenerative therapy. Mater Sci Eng, C 2012;32:2452–8.

[38] Bonab MM , Alimoghaddam K, Talebian F et al Aging of mesenchymal stem cell in vitro. BMC Cell Biol 7:14. BMC Cell Biol 2006;7:14. 16529651 10.1186/1471-2121-7-14 PMC1435883

[39] Legzdina D , Romanauska A, Nikulshin S et al Characterization of senescence of culture-expanded human adipose-derived mesenchymal stem cells. Int J Stem Cells 2016;9:124–36. 27426094 10.15283/ijsc.2016.9.1.124 PMC4961112

[40] Madl CM , Heilshorn SC. Engineering hydrogel microenvironments to recapitulate the stem cell Niche. Annu Rev Biomed Eng 2018;20:21–47. 29220201 10.1146/annurev-bioeng-062117-120954 PMC7266431

[41] Scadden DT. The stem-cell niche as an entity of action. Nature 2006;441:1075–9. 16810242 10.1038/nature04957

[42] Lee ST , Yun JI, Yun SJ et al Engineering integrin signaling for promoting embryonic stem cell self-renewal in a precisely defined niche. Biomaterials 2010;31:1219–26. 19926127 10.1016/j.biomaterials.2009.10.054

[43] Frith JE , Mills RJ, Hudson JE et al Tailored integrin–extracellular matrix interactions to direct human mesenchymal stem cell differentiation. Stem Cells Dev 2012;21:2442–56. 22455378 10.1089/scd.2011.0615 PMC3425135

[44] Saleh NT , Sohi AN, Esmaeili E et al Immobilized laminin-derived peptide can enhance expression of stemness markers in mesenchymal stem cells. Biotechnol Bioproc E 2019;24:876–84.

[45] Hynes RO. Integrins: bidirectional, allosteric signaling machines. Cell 2002;110:673–87. 12297042 10.1016/s0092-8674(02)00971-6

[46] Higuchi S , Watanabe TM, Kawauchi K et al Culturing of mouse and human cells on soft substrates promote the expression of stem cell markers. J Biosci Bioeng 2014;117:749–55. 24360205 10.1016/j.jbiosc.2013.11.011

[47] Chowdhury F , Li Y, Poh Y-C et al Soft substrates promote homogeneous self-renewal of embryonic stem cells via downregulating cell-matrix tractions. PLoS One 2010;5:e15655. 21179449 10.1371/journal.pone.0015655 PMC3001487

[48] Sun X , Zhang H, He J et al Adjustable hardness of hydrogel for promoting vascularization and maintaining stemness of stem cells in skin flap regeneration. Appl Mater Today 2018;13:54–63.

[49] Cheng N-C , Wang S, Young T-H. The influence of spheroid formation of human adipose-derived stem cells on chitosan films on stemness and differentiation capabilities. Biomaterials 2012;33:1748–58. 22153870 10.1016/j.biomaterials.2011.11.049

[50] Hsu SH , Huang GS, Feng F. Isolation of the multipotent MSC subpopulation from human gingival fibroblasts by culturing on chitosan membranes. Biomaterials 2012;33:2642–55. 22217805 10.1016/j.biomaterials.2011.12.032

[51] Wojtowicz AM , Shekaran A, Oest ME et al Coating of biomaterial scaffolds with the collagen-mimetic peptide GFOGER for bone defect repair. Biomaterials 2010;31:2574–82. 20056517 10.1016/j.biomaterials.2009.12.008 PMC2813962

[52] Shekaran A , García JR, Clark AY et al Bone regeneration using an alpha 2 beta 1 integrin-specific hydrogel as a BMP-2 delivery vehicle. Biomaterials 2014;35:5453–61. 24726536 10.1016/j.biomaterials.2014.03.055 PMC4033404

[53] Kim S , Cui ZK, Fan J et al Photocrosslinkable chitosan hydrogels functionalized with the RGD peptide and phosphoserine to enhance osteogenesis. J Mater Chem B 2016;4:5289–98. 28044100 10.1039/C6TB01154C PMC5200955

[54] Yang F , Williams CG, Wang DA et al The effect of incorporating RGD adhesive peptide in polyethylene glycol diacrylate hydrogel on osteogenesis of bone marrow stromal cells. Biomaterials 2005;26:5991–8. 15878198 10.1016/j.biomaterials.2005.03.018

[55] Darnell M , Young S, Gu L et al Substrate stress-relaxation regulates scaffold remodeling and bone formation in vivo. Adv Healthcare Mater 2017;6:1601185. 10.1002/adhm.201601185 PMC5440842 27995768

[56] Malcor J-D , Hunter EJ, Davidenko N et al Collagen scaffolds functionalized with triple-helical peptides support 3D HUVEC culture. Regen Biomater 2020;7:471–82. 33149936 10.1093/rb/rbaa025 PMC7597804

[57] Smith LA , Liu X, Jiang H et al The Enhancement of human embryonic stem cell osteogenic differentiation with nano-fibrous scaffolding. Biomaterials 2010;31:5526–35. 20430439 10.1016/j.biomaterials.2010.03.065 PMC2875265

[58] Sharma MB , Limaye LS, Kale VP. Mimicking the functional hematopoietic stem cell niche in vitro: recapitulation of marrow physiology by hydrogel-based three-dimensional cultures of mesenchymal stromal cells. Haematologica 2012;97:651–60. 22058199 10.3324/haematol.2011.050500 PMC3342965

[59] Salinas CN , Anseth KS. The influence of the RGD peptide motif and its contextual presentation in PEG gels on human mesenchymal stem cell viability. J Tissue Eng Regen Med 2008;2:296–304. 18512265 10.1002/term.95 PMC7842198

[60] Subramony SD , Dargis BR, Castillo M et al The guidance of stem cell differentiation by substrate alignment and mechanical stimulation. Biomaterials 2013;34:1942–53. 23245926 10.1016/j.biomaterials.2012.11.012 PMC3689925

[61] Schulz A , Katsen-Globa A, Huber EJ et al Poly(amidoamine)-alginate hydrogels: directing the behavior of mesenchymal stem cells with charged hydrogel surfaces. J Mater Sci Mater Med 2018;29:105. 29961123 10.1007/s10856-018-6113-x PMC6028859

