# Supplementary material for: Biomimetic cell-adhesive ligand-functionalized peptide composite hydrogels maintain stemness of human amniotic mesenchymal stem cells
Source: Regen Biomater. 2021 Mar 12;8(2):rbaa057. doi: 10.1093/rb/rbaa057 (PMC7953499; doi:10.1093/rb/rbaa057)
Supplement: rbaa057_Supplementary_Data [file rbaa057_supplementary_data.docx]

Supplementary Data

**Biomimetic cell-adhesive ligand-functionalized peptide composite hydrogels maintain stemness of human amniotic mesenchymal stem cells**

Ling Zhang, Na Xiong, Yanfei Liu*, Lili Gan

| **Table S1.** Oligonucleotide primers and PCR cycling conditions. | | | | |
| --- | --- | --- | --- | --- |
| Genes | GenBank  number | Primer sequences | Size  (bp) | Temp  (°C) |
| Oct4a | NM_001173531.2 | For: CAGAAGGGCAAGCGATCAAG  Rev: GAAAGGGACCGAGGAGTACAGTG | 171 | 60 |
| Nanog | NM_001297698.1 | For: CAACTGGCCGAAGAATAGCA  Rev: TCTGGTTGCTCCAGGTTGAA | 113 | 60 |
| Sox2 | NM_003106.3 | For: TGACCAGCTCGCAGACCTAC  Rev: TCGGACTTGACCACCGAAC | 112 | 60 |
| Rex1 | NM_174900.4 | For: AGAAACGGGCAAAGACAAGAC  Rev: GCTGACAGGTTCTATTTCCGC | 116 | 60 |
| Runx2 | NM_001015051.3 | For: TGGTTACTGTCATGGCGGGTA  Rev: TCTCAGATCGTTGAACCTTGCTA | 101 | 60 |
| Osx | NM_001173467.2 | For: ATCCAGCCCCCTTTACAAGC  Rev: TAGCATAGCCTGAGGTGGGT | 78 | 60 |
| BSP | NM_004967.3 | For: GGCACCAGTACCAACAGCAC  Rev: TTCCGGTCTCTGTGGTGTCTT | 124 | 60 |
| ALP | NM_001632.4 | For: ACTGGGGCCTGAGATACCC  Rev: TCGTGTTGCACTGGTTAAAGC | 185 | 60 |
| Ocn | NM_199173.5 | For: CCAGGCGCTACCTGTATCAA  Rev: GGTCAGCCAACTCGTCACAG | 111 | 60 |
| Col1α1 | NM_000088.3 | For: GAGGGCCAAGACGAAGACATC  Rev: CAGATCACGTCATCGCACAAC | 140 | 60 |
| Itgα2 | NM_002203.3 | For: CCTACAATGTTGGTCTCCCAGA  Rev: AGTAACCAGTTGCCTTTTGGATT | 106 | 60 |
| Itgα5 | NM_002205.4 | For: GGCTTCAACTTAGACGCGGAG  Rev: TGGCTGGTATTAGCCTTGGGT | 140 | 60 |
| Itgα6 | NM_001079818.2 | For: ATGCACGCGGATCGAGTTT  Rev: TTCCTGCTTCGTATTAACATGCT | 160 | 60 |
| Itgαv | NM_001145000.2 | For: ATCTGTGAGGTCGAAACAGGA  Rev: TGGAGCATACTCAACAGTCTTTG | 136 | 60 |
| Itgβ1 | NM_033668.2 | For: CCTACTTCTGCACGATGTGATG  Rev: CCTTTGCTACGGTTGGTTACATT | 128 | 60 |
| Itgβ5 | NM_002213.4 | For: TCTCGGTGTGATCTGAGGG  Rev: TGGCGAACCTGTAGCTGGA | 212 | 60 |
| GAPDH | NM_002046.4 | For: TCAAGAAGGTGGTGAAGCAGG  Rev: AGCGTCAAAGGTGGAGGAGTG | 119 | 60 |

| **Table S2.** Estimated structure fractions of the peptides in aqueous solution | | | | | | |
| --- | --- | --- | --- | --- | --- | --- |
| Peptide | Secondary structure fractions (%) | | | | | |
|  | H(r) | H(d) | S(r) | S (d) | Turn | Unrd |
| RADA-RGD in pure water | 0.06 | 0.06 | 0.25 | 0.14 | 0.25 | 0.23 |
| RADA-TTS in pure water | 0.00 | 0.04 | 0.22 | 0.13 | 0.21 | 0.42 |
| RADA-FOG in pure water | 0.08 | 0.06 | 0.28 | 0.16 | 0.28 | 0.13 |
| RADA16 in pure water | 0.08 | 0.07 | 0.27 | 0.15 | 0.27 | 0.15 |
| RADA-RGD in PBS (pH 7.2) | 0.01 | 0.05 | 0.22 | 0.13 | 0.21 | 0.38 |
| RADA-TTS in PBS (pH 7.2) | 0.00 | 0.05 | 0.19 | 0.11 | 0.19 | 0.45 |
| RADA-FOG in PBS (pH 7.2) | 0.01 | 0.05 | 0.24 | 0.14 | 0.23 | 0.34 |
| RADA16 in PBS (pH 7.2) | 0.03 | 0.06 | 0.22 | 0.13 | 0.22 | 0.33 |
| H (r), regular α-helix; H (d), distorted α-helix; S (r), regular β-strand; S (d), distorted β-strand (a partial but far-from-complete distortion of the regular β-strand, due to lack of some hydrogen bonds); Turn, β-turn structure; Unrd, unordered structure | | | | | | |

**Table S3.** Comparison of surface markers expression in composite hydrogels.

| Surface markers | Composite hydrogels | | | |
| --- | --- | --- | --- | --- |
|  | RADA16 | RGDmix | TTSmix | FOGmix |
| CD90 | 87.07±3.07 | 95.82±2.13** | 96.45±1.86** | 95.88±2.29** |
| CD105 | 91.65±4.24 | 93.66±2.61 | 94.61±1.74 | 92.17±2.30 |
| CD73 | 85.35±3.42 | 93.96±1.11** | 95.65±2.04** | 93.64±3.20** |
| CD44 | 90.03±5.44 | 96.51±1.86 | 95.54±1.68 | 97.05±1.19 |
| CD45/CD34/CD19  /CD11b/HLA-DR | 7.44±1.69 | 4.27±0.77 | 3.67±1.09* | 3.39±0.45* |

*P<0.05, **P<0.01 compared to RADA16.





**Figure S1.** Amino sequences of the peptides used in this study. The N- and C-termini of these peptides were blocked by acetylation and **amidation, respectively.**

**
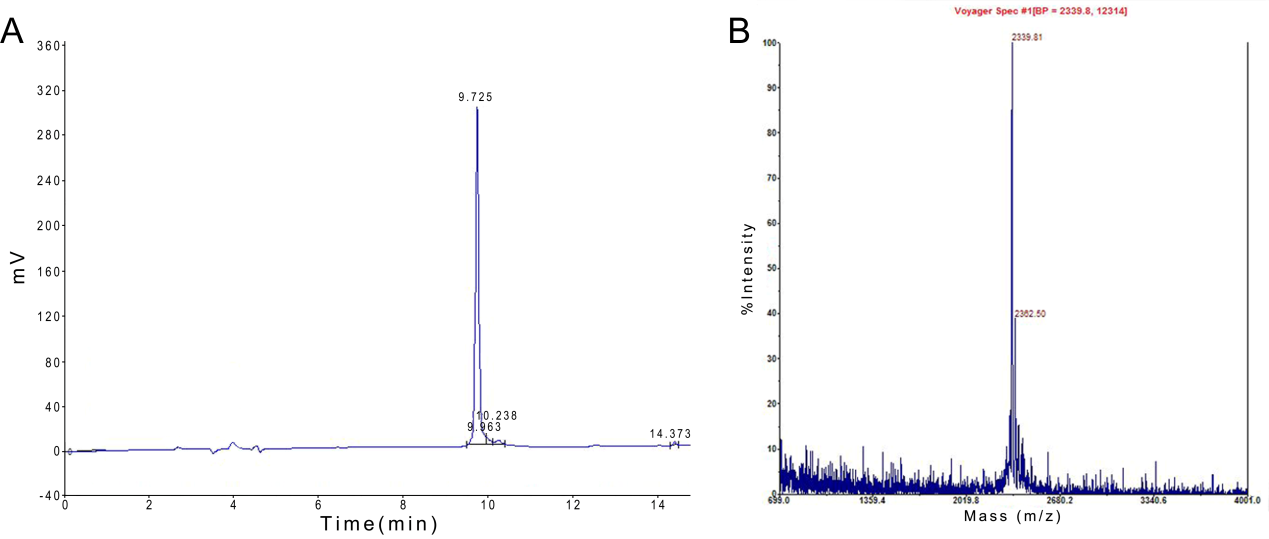
**

**Figure S2.** (A) HPLC chromatogram and (B) MALDI-TOF MS of peptide RADA16-RGD.


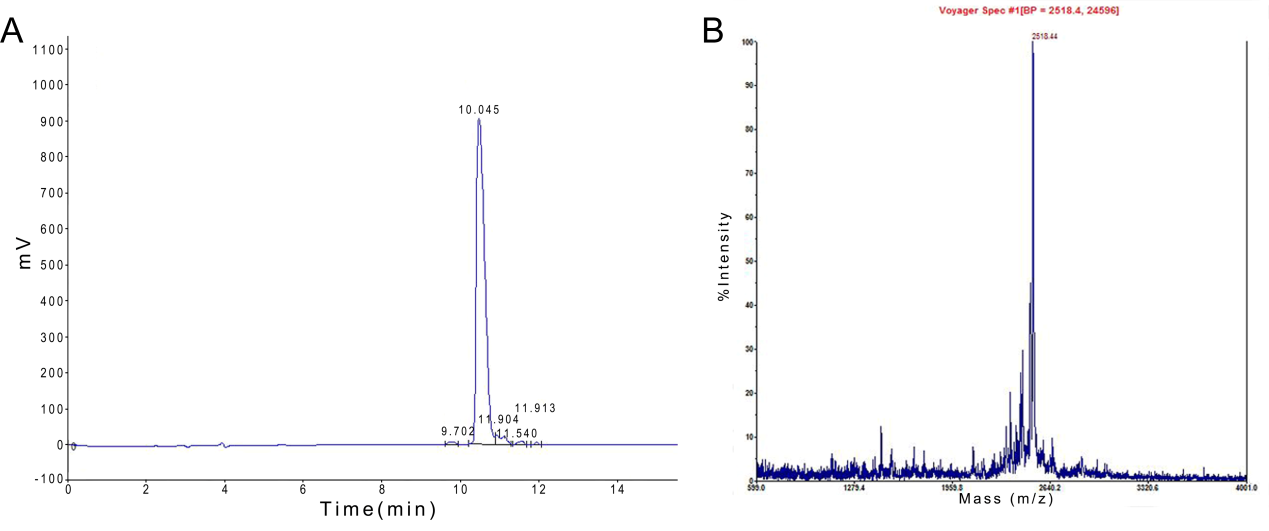


**Figure S3.** (A) HPLC chromatogram and (B) MALDI-TOF MS of peptide RADA16-TTS.


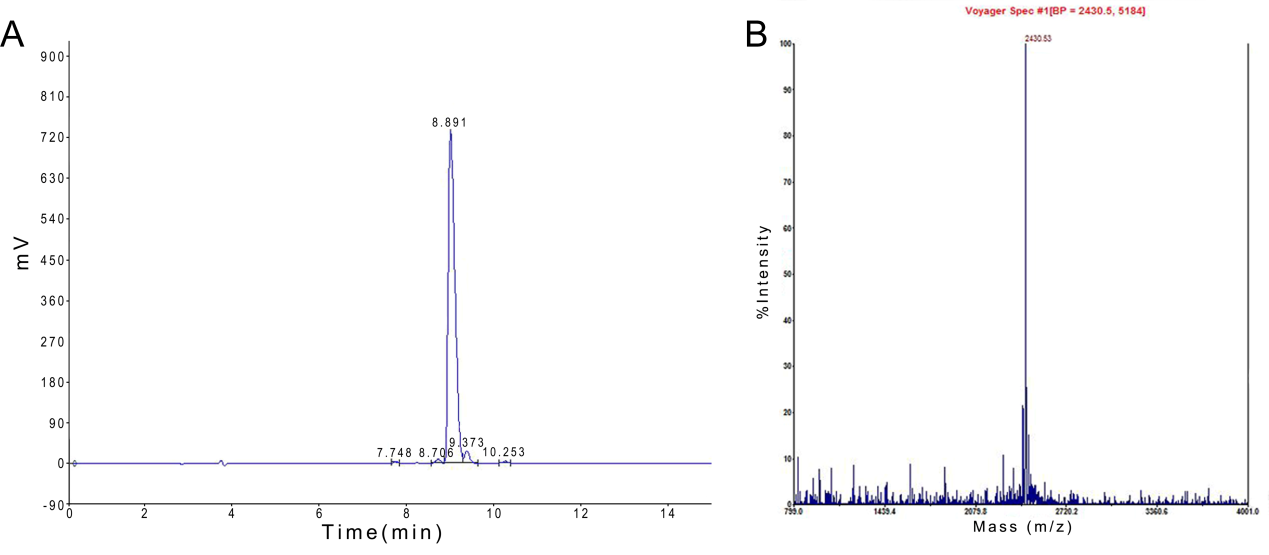


**Figure S4.** (A) HPLC chromatogram and (B) MALDI-TOF MS of peptide RADA16-FOG.


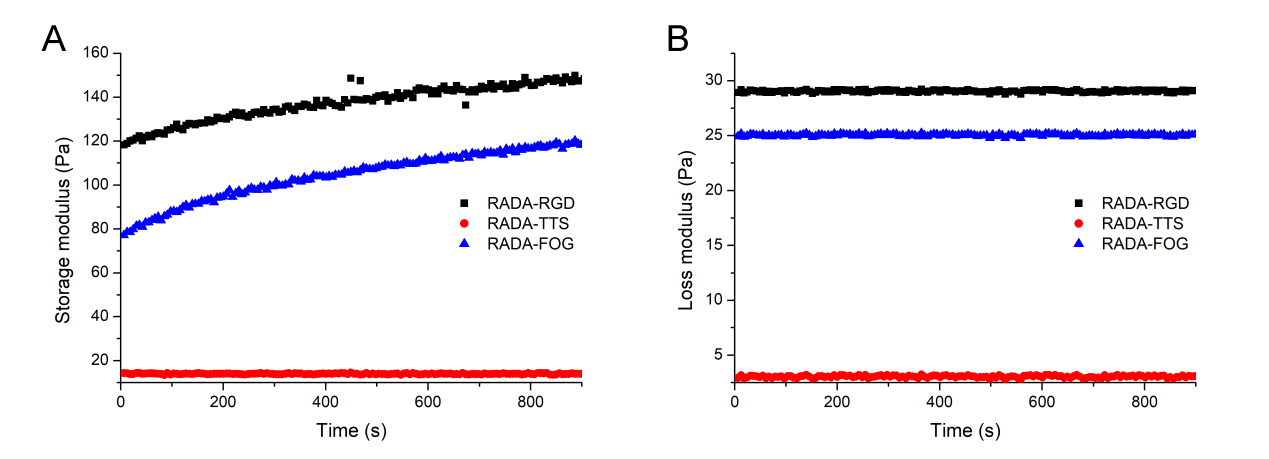


**Figure S5.** Rheology measurements of soft hydrogel formed by peptide RADA-RGD, RADA-TTS and RADA-FOG at 0.5% wt/vol. (A) Storage modulus and (B) loss modulus for the soft hydrogels. The hydrogels were made by mixing with an equal volume of PBS (pH 7.2).


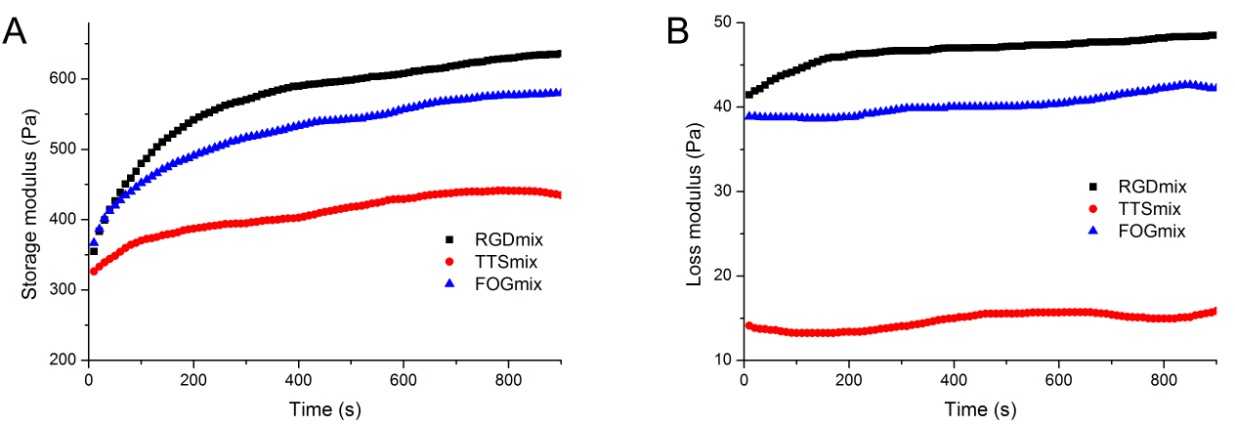


**Figure S6.** Rheology measurements of the composite hydrogels. Measure of (A) storage modulus and (B) loss modulus over time for the composite hydrogels. Peptide RADA-RGD, RADA-TTS and RADA-FOG (1% wt/vol) were separately mixed with RADA16 (1% wt/vol) at a ratio of 7:3 and followed by 20 min sonication. Then the composite hydrogels were made by mixing with an equal volume of PBS (pH 7.2). The final concentration of the peptides were 0.5% wt/vol.


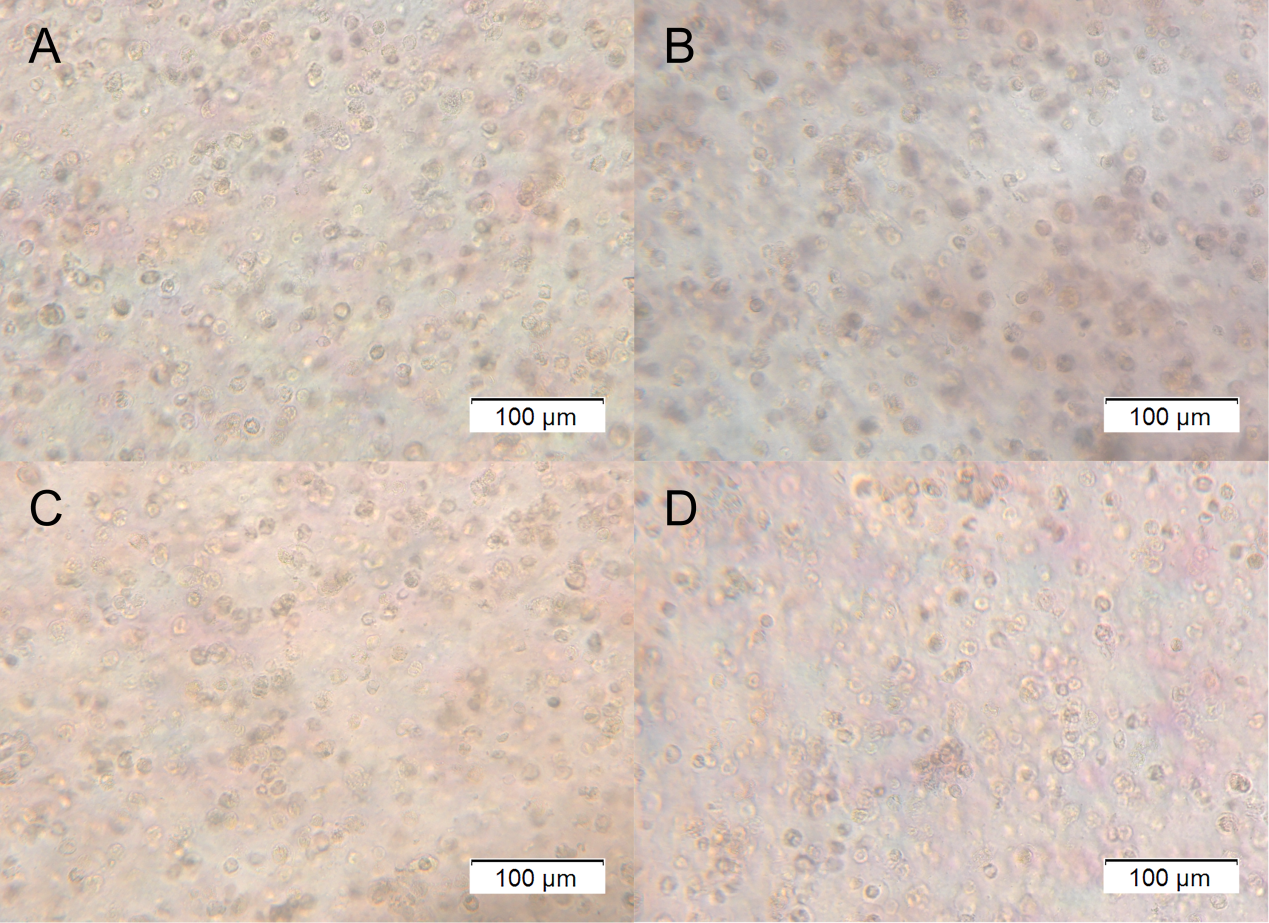


**Figure S7.** The visual images of the hAMSCs encapsulated in the hydrogel (A) RADA16, (B) RGDmix, (C) TTSmix and (D) FOGmix after 1 days culture.


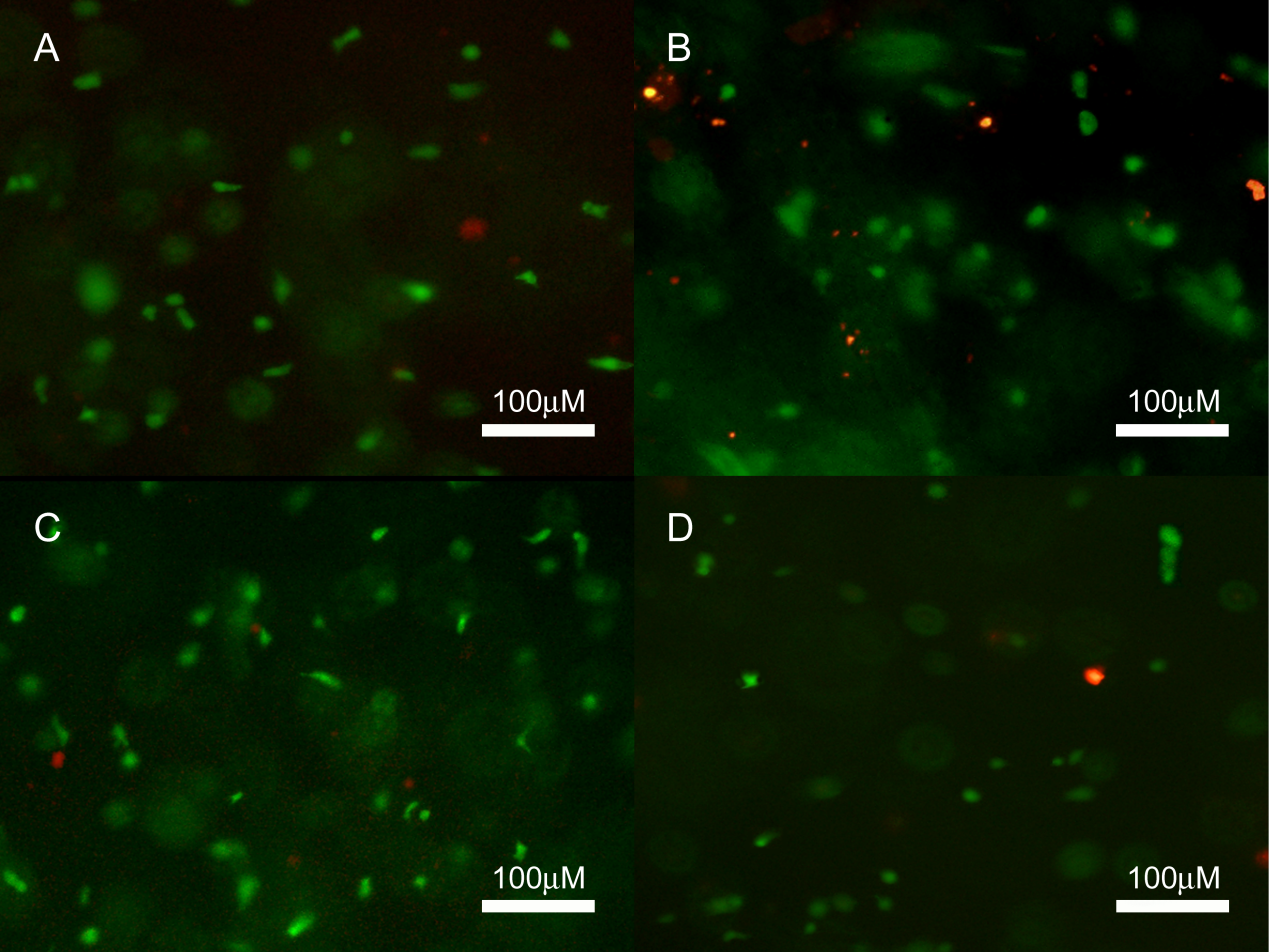


**Figure S8.** Fluorescence microscopy images of hAMSCs stained as live (Calcein AM/Green) or dead (Ethidium homodimer-1/Red) after 3 days of culture while encapsulated in hydrogel (A) RADA16, (B) RGDmix, (C) TTSmix and (D) FOGmix.
